# Evaluation of a Lateral Flow Assay for Rapid Detection of African Swine Fever Virus in Multiple Sample Types

**DOI:** 10.3390/pathogens11020138

**Published:** 2022-01-24

**Authors:** Chukwunonso Onyilagha, Kelvin Nguyen, Pam D. Luka, Ularamu Hussaini, Adeyinka Adedeji, Theophilus Odoom, Aruna Ambagala

**Affiliations:** 1 National Centre for Foreign Animal Disease, Canadian Food Inspection Agency, Winnipeg, MB R3E 3M4, Canada; chukwunonso.onyilagha@inspection.gc.ca (C.O.); nguyenk9@myumanitoba.ca (K.N.); 2 Virology Division, National Veterinary Research Institute, P.M.B 01, Vom 930001, Plateau, Nigeria; pamluka08@gmail.com (P.D.L.); ularamuhussaini@yahoo.co.uk (U.H.); yinkadeji@yahoo.com (A.A.); 3 Accra Laboratory, Veterinary Services Directorate, Accra P.O. Box M161, Ghana; theodoom@yahoo.com; 4 Department of Comparative Biology, Faculty of Veterinary Medicine, University of Calgary, Calgary, AB T2N 1N4, Canada

**Keywords:** African swine fever, lateral flow assay, PenCheck, real-time PCR

## Abstract

Antibody-based lateral flow assay (LFA) is a quick and inexpensive tool used to detect pathogens in field samples, especially in hard-to-reach remote areas that may have limited access to central laboratories during an outbreak or surveillance. In this study, we investigated the ability of a commercially available LFA, PenCheck^®^, to detect African swine fever virus (ASFV) in clinical samples derived from pigs infected with highly virulent ASFV strains. The assay was specific and positively identified the majority of pigs showing high fever during the early stages (between 3 and 5 days) of infection. PenCheck^®^ LFA also detected ASFV in serum and tissue samples collected from pigs that succumbed to experimental ASFV infection and whole blood, plasma, and tissue samples from the field. The limit of detection of the assay was ASFV titer 10^7.80^ TCID_50_/mL, corresponding to ASFV real-time PCR values below 23 Ct. Although the sensitivity of the assay is less than that of the laboratory-based real-time PCR assays, the results obtained with the PenCheck^®^ LFA in this study suggest that it can be used as a herd-level, field-deployable, and easy-to-use diagnostic tool to identify ASF-affected farms when access to portable molecular assays or central laboratories is not possible.

## 1. Introduction

African swine fever (ASF) remains a global priority transboundary animal disease. It is a highly fatal hemorrhagic disease of domestic and wild swine that continues to spread across Africa, Europe, Asia, and the Caribbean [1,2,3,4,5,6,7,8,9]. The causative agent, ASF virus (ASFV), is a structurally complex, large double-stranded DNA virus and the sole member of the Asfaviridae family belonging to the genus Asfivirus [10]. ASF, a reportable disease to the World Organization for Animal Health (OIE), can cause up to 100% mortality depending on the strain. The transmission of ASFV can occur through direct contact between infected and uninfected pigs or indirectly through contaminated meat products, fomites, or tick bites [11]. Until recently, ASF remained endemic in Africa [12,13] and Sardinia [7,14]; however, in 2007, ASF spread to Georgia, then to Russia, eastern and western Europe, Southeast Asia, and the Caribbean, killing millions of pigs with accompanying severe economic losses [9,11,13,15,16,17]. There is currently no commercially available approved vaccine for ASF [18,19]. Therefore, early detection is critical for controlling the spread of the virus. Real-time PCR is the most widely used method for detecting ASFV genomic material in clinical samples [20]. The current real-time PCR assays are highly sensitive and specific, but they are generally laborious, expensive, and require trained personnel in the laboratory to conduct the testing. Other molecular assays, including portable real-time PCR assays and loop-mediated isothermal amplification (LAMP) assays, have been developed for field testing; however, they still demand some technical competencies at the field level as they require nucleic acid extraction and pipetting [21,22,23,24,25,26,27,28,29,30,31].

Lateral flow assays (LFAs), in contrast, can easily be performed by following simple instructions, and are thus ideal for field use. Many LFAs have been developed and commercialized for pen/bedside use to detect biomarkers, viral and bacterial antigens in various clinical sample types like whole blood, serum, plasma, urine, and saliva [32,33,34,35]. Recently, an antibody-based LFA, PenCheck^®^ (Silver Lake Research, CA, USA), was developed and commercialized in the USA for portable detection of ASF. Here, we describe the evaluation of PenCheck^®^ LFA for its ability to detect African swine fever virus (ASFV) in clinical samples collected from pigs infected with highly virulent ASFV strains. PenCheck^®^ LFA targets ASFV P30, a highly antigenic, secreted phosphoprotein expressed during the early stages of ASFV infection [36].

## 2. Results and Discussion

There are highly sensitive and specific molecular assays available for ASFV genome detection. Most of these assays demand sophisticated and expensive instrumentation and highly skilled staff; therefore, they are mostly limited to laboratory use. In recent years, some of these laboratory-based molecular assays have been adapted for use on portable devices [37,38]. Novel assays, such as LAMP, clustered regularly interspaced short palindromic repeats (CRISPR)/Cas12a, and recombinase polymerase amplification (RPA) assays, have been developed for field detection of ASF [21,22,23,24,25,26,27,28,29,30,31]. These assays, however, can be costly and demand some technical expertise. Therefore, portable molecular assays may not be useful for ASF diagnosis in poor resource settings.

Antibody-based LFAs, in contrast, are relatively cheap, require no additional instrumentation, and can easily be performed by anyone following simple instructions. They are one-time-use assays and pose no risk of transmitting pathogens between farms when used during an outbreak [25,26,39]. In general, antibody-based LFAs are less sensitive compared with their molecular counterpart assays. However, as pigs infected with highly virulent ASFV strains develop high viral titers in blood within a few days after infection, antibody-based LFAs could be useful for rapidly detecting ASFV-infected pigs during the early stages of the infection. Therefore, we wanted to determine the suitability of the commercially available antibody-based LFA, PenCheck^®^, for early detection of ASFV in pigs infected with highly virulent ASFV strains.

Towards this goal, we first determined the analytical sensitivity of the PenCheck^®^ LFA using a dilution series of whole blood samples collected from two pigs infected with highly virulent ASFV strains, ASFV Georgia 2007/1 (Pig #134) or ASFV Nigeria RV502 (Pig #2). For comparison, the serial dilutions were also subjected to real-time PCR and virus titration. PenCheck^®^ LFA detected ASFV in undiluted and tenfold-diluted whole blood samples (Table 1 (A,B)) for both viruses, translating to Ct values of <23 and virus titers ≥10^7.80^ (Table 1 (A)) and ≥10^7.89^ (Table 1 (B)) TCID_50_/mL. To assess the analytical specificity of the PenCheck^®^ LFA, we used archived cell culture-amplified ASFV p72 genotype I strains (ASFV Malta’78, ASF OURT/88/3, and ASFV Malawi LIL 18/2) and whole blood and tissue (spleen) samples collected from pigs infected with three different classical swine fever virus (CSFV) strains, as well as other cell culture-amplified porcine viruses. PenCheck^®^ LFA detected all ASFV p72 genotype I strains tested and did not cross-react with any tested classical swine fever (CSF), vesicular stomatitis, foot-and-mouth disease, swine vesicular disease, and Seneca valley virus strains (Table 1 (C)). To evaluate the reproducibility of the assay, three known ASFV real-time PCR-positive samples (pig #140, #141, and #145, infected with ASFV Georgia 2007/1) and one known negative (ASFV uninfected) whole blood sample were tested ten times using the PenCheck^®^ LFA. The results were consistent across the samples assessed (Table 1 (D)). The real-time PCR-negative (clean blood) and positive (with a Ct value of 23.6) whole blood samples were not detected by PenCheck^®^ LFA, consistent with our earlier observation.

Currently, there is no effective vaccine for ASF; therefore, rapid detection, preferably on-site, and removal of the infected and contact pigs (stamping out) is the most effective approaches to curtail the spread of the disease. Highly virulent strains of ASFV have an incubation period of 2–3 days; fever, lack of appetite, and lethargy are the first clinical signs to appear. Fever in ASFV-infected animals coincides with viremia, which quickly peaks (within 1–2 days of fever) up to 10^8–9^ TCID_50_/mL [40,41]. Therefore, despite the low sensitivity of the PenCheck^®^ LFA compared with real-time PCR (see Table 1 (A,B)), we wanted to determine if the PenCheck^®^ LFA can be used to detect ASFV in whole blood samples collected from pigs showing early clinical signs of ASF. For this, we used whole blood samples collected from pigs directly or indirectly (contact with a directly infected pig) infected with two different strains of highly virulent ASFV (ASFV Georgia 2007/1 and ASFV Nigeria RV502) belonging to p72 genotype II. The PenCheck^®^ LFA results were compared with the real-time PCR results and rectal temperatures. Pigs with rectal temperatures above 40 °C for two consecutive days were considered as having a fever.

The first set of whole blood samples tested were collected from ASFV Georgia 2007/1 infected pigs (Figure S2). Whole blood samples collected from a seeder pig (#138) that was directly infected with ASFV Georgia 2007/1 tested positive on PenCheck^®^ LFA starting from 4 dpi, the same day as the onset of fever (real-time PCR Ct value of 21.6) (Table 2). The seeder pig remained positive on PenCheck^®^ LFA until it was found dead on 7 dpi. Four contact pigs (#126, 129, 139, and 141) started to develop fever 10 days post-exposure (dpe); two of them (#139 and 141) tested positive on PenCheck^®^ LFA on 12 dpe. Three additional pigs (#144, 145, and 146) started to develop fever on 12 dpe; in addition to pigs #139 and 141, two of those three pigs (#145 and 146) tested positive on PenCheck^®^ LFA on 12 dpe. On 13 dpe, two more pigs (#130 and 140) developed fever, and three pigs (#126, 132 and 134) tested positive on PenCheck^®^ LFA. The remaining four pigs that developed fever tested positive on PenCheck^®^ LFA on 14 (pigs #140 and 144) and 15 dpe (pigs #129 and 130). One contact pig (#133) remained uninfected (based on Ct value and fever data) and tested negative on PenCheck^®^ LFA until the termination of the experiment on 17 dpe. The remaining animals tested positive on PenCheck^®^ LFA at least once during the study when their whole blood Ct values fell below 23. Two samples (pig #130, 17 dpe and pig #134, 16 dpe), however, repeatedly tested negative on PenCheck^®^ LFA despite having low Ct values (20.9 and 21.6, respectively). In order to determine the cause of the negative results, the viral titers of these two samples were determined to be 10^9.10^ and 10^5.87^ TCID_50_/mL for pigs #130 on 17 dpe and pig #134 on 16 dpe, respectively. While the low ASFV titer in the sample from pig #134 could account for the negative results obtained with PenCheck^®^ LFA, the same could not be said of the sample from pig #130. We speculate that the discordance could be due to additional factors specific to these two samples.

In pigs, rectal temperature starting at 40.5 °C is considered a high fever, and at this point, they often become lethargic and show less interest in feeding. All pigs that developed a high fever for two consecutive days in this experiment had whole blood Ct values below 23, and when tested, they were identified as ASF positive by the PenCheck^®^ LFA.

To further evaluate PenCheck^®^ LFA, we used whole blood and tissue samples collected from two pigs simultaneously infected with ASFV Nigeria RV502, an isolate obtained from the 2020 ASF p72 genotype II outbreak in Nigeria (Table 3). Of the two pigs, #2 started to develop fever on 3 dpi, and #1 on 4 dpi. Pig #1 tested positive by real-time PCR on 3 dpi and #2 on 4 dpi. Both animals tested positive on PenCheck^®^ LFA on 4 dpi, when they developed a high fever (≥40.5 °C) and remained positive until they were found dead (pig #1) or euthanized (pig #2) on 6 dpi. When pig #2 tested positive on PenCheck^®^ LFA, the whole blood sample had a Ct value of 27.12. The same sample was tested multiple times on PenCheck^®^ LFA and by real-time PCR, and the results remained the same.

In addition to the whole blood samples, serum, nasal, oral, and rectal swabs collected from these two animals were tested on PenCheck^®^ LFA. All nasal, oral, and rectal swabs had real-time PCR Ct values above 23; therefore, they tested negative on PenCheck^®^ LFA. The two serum samples, pig #1, 4 dpi and Pig #2, 6 dpi, with Ct values of 21.01 and 18.74, respectively, tested positive on the PenCheck^®^ LFA. In addition to ante-mortem samples, a number of post-mortem samples (lymph nodes, tonsils, and spleen) collected from pigs #1 and 2 were tested on the PenCheck^®^ LFA as well. All samples tested positive on PenCheck^®^ LFA and had a high ASFV genome load (Ct values ranging from 17.12 to 22.05) (Table 3 (B)).

To assess the performance of PenCheck^®^ LFA with field samples, we tested whole blood, plasma, and spleen samples collected from domestic pigs on several farms in different geographical regions in Ghana and Nigeria. The samples were first tested for ASFV genomic material using the NCFAD laboratory-based ASFV real-time PCR assay to obtain their Ct values; out of the 50 field samples tested by real-time PCR, a mixture of strong positive, weak positive, and suspicious samples were identified and subjected to testing using PenCheck^®^ LFA. One sample with Ct value lower than 23 unexpectedly tested negative on PenCheck^®^ LFA (Table 4 (A)). Surprisingly, a few samples with Ct values higher than 23 and those deemed suspicious by real-time PCR assay also tested positive on PenCheck^®^ LFA. While the few samples mentioned above did not strictly adhere to the predetermined LOD for PenCheck^®^ LFA, the observations could be due to the degradation of ASFV nucleic acid caused by poor and/or prolonged storage conditions of some of the field samples. For clinical specificity, we also tested 179 known ASFV-negative whole blood samples from Canada (79) and Ghana (100). All the samples were confirmed to be negative for ASFV genomic material by ASFV real-time PCR assay. All the samples except one sample from Ghana tested negative on PenCheck^®^ LFA, demonstrating 99.4% specificity (Table 4 (B)).

The two discordant field samples (Table 4 (A,B)) mentioned above were further tested using a different LFA, INgezim^®^ ASF CROM Ag (Eurofins Technologies Ingenasa, Madrid, Spain), and the results remained unchanged (data not shown). While the real-time PCR-positive, but INgezim^®^ ASF CROM Ag and PenCheck^®^ LFA-negative sample could be explained by poor sample quality, the PenCheck^®^ LFA-positive, but real-time PCR and INgezim^®^ ASF CROM Ag LFA-negative sample could be a true false-positive detection (although with a faint positive test band) by PenCheck^®^ LFA.

To the best of our knowledge, INgezim^®^ ASF CROM Ag is the only other commercially available antibody-based LFA for ASF detection [42]; it targets VP72, a highly immunogenic capsid protein of ASFV, and has shown 100% specificity. However, it only detected pigs with high viral loads corresponding to 4–7 dpi. The assay was recently evaluated for its ability to detect ASFV in whole blood and serum samples collected from experimentally infected domestic and wild boars, and wild boars that were naturally infected with highly virulent ASFV strains [43]. The INgezim^®^ ASF CROM Ag detected ASFV in the majority of the whole blood samples collected from experimentally infected domestic pigs and wild boars with Ct values up to 29. The assay, however, failed to detect a few blood samples from the experimentally infected animals with Ct values as low as 20. When the assay was tested using field-collected wild boar samples, its sensitivity was drastically reduced, likely due to the poor quality of the field samples. The sensitivity of the INgezim^®^ ASF CROM Ag LFA improved when the samples were subjected to a freeze–thaw cycle, possibly owing to the release of ASFV antigens bound to the red blood cells in the sample. However, we observed no difference in our results (sensitivity or specificity) after the freeze–thaw cycle before the samples were tested on the PenCheck^®^ LFA (data not shown). The PenCheck^®^ LFA protocol includes a 5 min lysis step at room temperature before the sample comes into contact with the lateral flow strip. This may explain the above observation.

In summary, we showed that the commercially available PenCheck^®^ LFA could identify ASFV-infected animals showing a high fever, which coincides with high viral titers that are commonly observed between 3 and 5 dpi with highly virulent ASFV strains. We also showed that PenCheck^®^ LFA could correctly identify most field samples collected from ASFV-infected domestic pigs. In addition to whole blood, the assay can also detect ASFV antigens in other clinical samples such as serum and tissues collected from pigs that succumbed to ASFV infection. Pigs that die of highly virulent ASF infections have high viral loads in their internal organs, such as the spleen, hemorrhagic lymph nodes, and tonsils. Therefore, it is conceivable that PenCheck^®^ LFA would be suitable for detecting ASFV antigens in most of those samples. Owing to low viral loads, sample types such as nasal, oral, and rectal swabs are not suitable for testing using the PenCheck^®^ LFA.

In this study, we only used clinical samples collected from pigs infected with highly virulent ASFV strains. However, using cell culture-amplified ASFV Malta’78 and OURT/88/3 strains, we showed that the PenCheck^®^ LFA is able to detect moderately and low virulent ASFV strains, respectively. Similar to highly virulent strains, moderately virulent ASFV strains also induce high levels of viremia in infected animals, with a slower disease progression. Therefore, it is conceivable that PenCheck^®^ LFA would detect pigs presenting fever and high viremia due to infection with moderately virulent ASFV strains. However, the same cannot be said regarding low virulent ASFV strains as the infected animals seldomly present fever or high viremia; therefore PenCheck^®^ LFA is highly unlikely to detect ASFV in pigs infected with low virulent ASFV strains.

In conclusion, our data showed that, although not as sensitive as molecular assays, the PenCheck^®^ LFA would be a valuable tool for herd-level pen-side detection of ASFV in pigs displaying moderate to severe clinical signs, where access to molecular assays is not feasible. Further improvement of sensitivity of the assay would broaden its use in future ASF control efforts.

## 3. Materials and Methods

### 3.1. Lateral Flow Assay

The PenCheck^®^ LFA kit consists of test strips and test vials containing lyophilized reagents. Whole blood, serum, swabs, and tissues were tested according to the manufacturer’s recommendations. Briefly, 200 µL of double-distilled water was added to the test vial containing lyophilized reagents, followed by 10 µL of the sample. The tube was gently shaken by hand to mix the contents. After 5 min of incubation at room temperature, the test vial was gently swirled, and the test strip was inserted into the vial. Incubation continued for an additional 20 min, after which it was removed and examined by the naked eye for the appearance of lines. One line (control) indicates a negative sample, and two lines (control and test) indicate a positive sample (Figure S1).

### 3.2. Clinical Samples from Experimentally Infected Animals

Clinical samples used in this study were obtained from pigs experimentally infected with different ASFV strains at the National Centre for Foreign Animal Disease (NCFAD) or from pigs naturally infected with ASFV field strains in Ghana and Nigeria.

ASFV Georgia 2007/1 (p72 genotype II)-positive whole blood samples were obtained from a recent animal experiment conducted at the NCFAD to evaluate the potential use of oral fluid samples for early detection of ASFV in commercial herds [44]. In this experiment, 1 (pig #138) out of 24 pigs in the pen was infected with 1 × 10^5^ TCID_50_ of ASFV Georgia 2007/1 (in 1 mL) intramuscularly and returned to the pen afterwards. The animals were monitored twice a day for clinical signs, and their rectal temperatures were recorded daily. In addition to pen-based daily oral fluid samples, whole blood, nasal, or oropharyngeal swabs from each animal were collected every other day. To evaluate the PenCheck^®^ LFA, whole blood samples collected from the seeder pig (pig #138) and 12 other randomly selected contact pigs that developed clinical signs during the early phase of the infection were used.

To obtain additional whole blood and tissue samples, two pigs (pigs 1 and 2) were infected with ASFV RV502, an ASFV p72 genotype II isolate from the 2020 ASF outbreak in Nigeria [45], through the oronasal route (1X10^5^ TCID_50_ in 3 mL, 2 mL orally, and 0.5 mL per nostril). After infection, both animals were monitored twice daily for clinical signs and rectal temperatures were recorded daily. In addition to the whole blood samples, different tissue samples (hemorrhagic lymph nodes, tonsils, spleen, and diaphragm) were collected from the two pigs’ post-mortem after they succumbed to the infection (5 dpi for pig #1 and 6 dpi for pig #2). In both of these experiments, four- to five-week-old Landrace-Large White cross-bred grower pigs purchased from a local farm in Winnipeg, Manitoba, Canada, were used.

For specificity testing, three additional strains of cell culture amplified ASFV strains, Malta’78 and OURT/88/3 (p72 genotype I) and Malawi LIL18/2 (genotype II), and archived whole blood and spleen samples collected from pigs experimentally infected with three classical swine fever virus (CSFV) strains, Kanagawa (2 pigs), Diepholz (4 pigs), and Koslov (3 pigs), were used. In addition, archived tissue culture-propagated Vesicular stomatitis virus—NJ and IN, foot-and-mouth disease virus—OUKG 11/01 and A24 Cruzeiro, Swine vesicular disease virus UK27/72, and Seneca Valley virus (SVV) were tested.

All ASFV strains used in this study were propagated in primary porcine peripheral leukocytes and tittered on porcine alveolar macrophages as described previously [46].

### 3.3. Clinical Samples from Field Outbreaks

The field samples (all from domestic pigs) used in this study were either obtained as part of an ongoing surveillance program in Ghana or collected during the 2020 ASF outbreak in Nigeria. The whole blood and plasma samples were directly tested as received, while the spleen samples were first homogenized to produce 10% w/v suspensions before testing.

### 3.4. Nucleic Acid Extraction and Real-Time Reverse Transcription-Polymerase Chain Reaction

The total nucleic acids extraction was performed from samples using the MagMAX™ Pathogen RNA/DNA Kit and the MagMAX Express-96 Magnetic Particle Processor (Life Technologies, Burlington, ON, Canada) following the manufacturer’s recommended protocol. The tissue samples used were first homogenized to prepare 10% w/v suspensions before nucleic acid extraction, and the whole blood samples were directly used without further processing.

A previously published TaqMan qPCR assay that specifically amplifies a conserved region of the p72 gene of ASFV was used to quantify the ASFV genomic material [47]. The TaqMan™ Fast Virus 1-Step master mix (Life Technologies, Burlington, ON, Canada) was used to perform the real-time PCR on Bio-Rad CFX96 Touch™ (Bio-Rad, Hercules, CA, USA) using the recommended cycling conditions (50 °C for 10 min, 95 °C for 3 min, followed by 40 cycles of 96 °C for 3 s and 60 °C for 30 s). The TaqMan qPCR assay for beta-actin [48] was used as the internal control for optimal nucleic acid extraction and the absence of PCR inhibitors in all assessed samples. Cycle threshold (Ct) values of 35.99 and lower were considered positive, and values between 36 and 40 were deemed suspicious and repeated.

## Figures and Tables

**Table 1:** Sensitivity, specificity, and reproducibility of the PenCheck^®^ LFA.

**A**							
**ASFV Georgia 2007/1, Pig 134**	**Ct Value (Run 1)**		**Ct Value (Run 2)**		**PenCheck (Run 1)**	**PenCheck (Run 2)**	**ASFV Titer (TCID_50_/mL)**
	**ASFV**	**β-Actin**	**ASFV**	**β-Actin**			
Neat	19.22	23.43	20.08	23.12	Pos	Pos	10^8.87^
10^−1^	22.63	24.67	22.66	24.88	Pos	Pos	10^7.80^
10^−2^	25.64	26.01	25.57	26.34	Neg	Neg	10^6.71^
10^−3^	28.24	25.23	27.88	25.95	Neg	Neg	nd
10^−4^	30.89	26.34	31.21	26.29	Neg	Neg	nd
**B**							
**ASFV Nigeria RV502, Pig 2**	**Ct Value (Run 1)**		**Ct Value (Run 2)**		**PenCheck (Run 1)**	**PenCheck (Run 2)**	**ASFV Titer (TCID50/mL)**
	**ASFV**	**β-Actin**	**ASFV**	**β-Actin**			
Neat	19.42	22.22	18.66	22.02	Pos	Pos	10^9.1^
10^−1^	22.01	24.63	22.01	24.54	Pos	Pos	10^7.89^
10^−2^	25.11	26.90	24.82	26.61	Neg	Neg	10^6.80^
10^−3^	27.71	27.36	27.23	27.16	Neg	Neg	10^4.87^
10^−4^	29.52	26.71	29.09	26.54	Neg	Neg	10^3.87^
**C**							
**Sample**						**Ct Value**	**PenCheck**
ASFV Malta’78						nd	Pos
ASFV OURT/88/3						nd	Pos
ASFV Malawi LIL 18/2						nd	Pos
CSFV Kanagawa, P2, DPI 42						16.0	Neg
CSFV Kanagawa, P4, DPI 42						19.4	Neg
CSFV Diepholz, P3, DPI 8-2						22.1	Neg
CSFV Diepholz, P1, DPI 12–6						22.1	Neg
CSFV Diepholz, P2, DPI 12–6						20.5	Neg
CSFV Diepholz, P3, DPI 12–6						21.1	Neg
CSFV Diepholz, P4, DPI 12–6						22.2	Neg
CSFV Koslov, P5, DPI 6–8, Spleen						16.4	Neg
CSFV Koslov, P7, DPI 6–8, Spleen						18.4	Neg
CSFV Koslov, P8, DPI 6–8, Spleen						17.6	Neg
Vesicular Stomatitis Virus-NJ						15.01	Neg
Vesicular Stomatitis Virus-IN						15.29	Neg
Foot-and-mouth Disease Virus OUKG 11/01						12.36	Neg
Foot-and-mouth disease virus A24 Cruzeiro						11.99	Neg
Swine Vesicular Disease Virus UK27/72						11.61	Neg
Seneca Valley Virus						12.7	Neg
**D**							
	**PenCheck** **: ASFV Georgia 2007/1**						
	**Pig 140, DPI-12 (Ct: 23.6)**		**Pig 141, DPI-12 (Ct: 19.9)**		**Pig 145, DPI-12 (Ct: 21.0)**		**Clean Blood (Ct: 0.0)**
Run 1	Neg		Pos		Pos		Neg
Run 2	Neg		Pos		Pos		Neg
Run 3	Neg		Pos		Pos		Neg
Run 4	Neg		Pos		Pos		Neg
Run 5	Neg		Pos		Pos		Neg
Run 6	Neg		Pos		Pos		Neg
Run 7	Neg		Pos		Pos		Neg
Run 8	Neg		Pos		Pos		Neg
Run 9	Neg		Pos		Pos		Neg
Run 10	Neg		Pos		Pos		Neg

The analytical sensitivity of the assay was measured using serial dilution of blood samples collected from pigs infected with ASFV Georgia 2007/1 (**A**) or ASFV Nigeria RV502 (**B**). The specificity of the PenCheck^®^ LFA was measured using cell culture-amplified ASFV strains (genotype I) and whole blood and tissue samples from pigs infected with classical swine fever virus strains, as well as cell culture-amplified vesicular stomatitis virus strains, foot-and-mouth disease virus strains, swine vesicular disease virus, and Seneca valley virus (**C**). Reproducibility of the assay was measured using blood samples collected from three pigs infected with ASFV Georgia 2007/1 (**D**). For (**C**), the β-actin Ct value range for the samples tested by real-time PCR was 19.4 to 25.1. Pos, positive (green); Neg, negative (yellow); nd, not done.

**Table 2:** Comparing PenCheck^®^ LFA results with the onset of clinical sign (fever) and detection of ASFV genomic material in ASFV Georgia 2007/1-infected pigs.

	**DPI 2**			**DPI 4**			**DPI 5**			**DPI 6**																				
Pig #	Temp.	Ct	PenCheck	Temp.	Ct	PenCheck	Temp.	Ct	PenCheck	Temp.	Ct	PenCheck																		
Pig 138	na	38.1	Neg	40.8	21.6	Pos	40.6	21.1	Pos	40.8	20.6	Pos																		
	**DPE 8**			**DPE 9**			**DPE 10**			**DPE 11**			**DPE 12**			**DPE 13**			**DPE 14**			**DPE 15**			**DPE 16**			**DPE 17**		
Pig #	Temp.	Ct	PenCheck	Temp.	Ct	PenCheck	Temp.	Ct	PenCheck	Temp.	Ct	PenCheck	Temp.	Ct	PenCheck	Temp.	Ct	PenCheck	Temp.	Ct	PenCheck	Temp.	Ct	PenCheck	Temp.	Ct	PenCheck	Temp.	Ct	PenCheck
Pig 126	39.7	na	na	39.7	0.0	Neg	40.0	na	na	40.4	28.9	Neg	40.9	na	na	40.5	20.1	Pos	41.3	na	na									
Pig 129	39.8	na	na	39.6	0.0	Neg	40.0	na	na	40.1	0.0	Neg	39.9	na	na	41.6	25.3	Neg	41.2	na	na	41.6	20.9	Pos	41.6	na	na	40.7		
Pig 130	39.4	na	na	39.3	na	na	38.7	na	na	39.6	0.0	Neg	39.5	na	na	41.0	27.9	Neg	41.1	na	na	41.5	19.1	Pos	40.6	na	na	41.7	20.9	Neg
Pig 132	39.6	na	na	38.9	0.0	Neg	39.0	na	na	39.9	24.2	Neg	41.4	na	na	41.3	19.8	Pos												
Pig 133	39.5	na	na	39.1	na	na	39.7	na	na	39.7	na	na	39.8	na	na	39.8	0.0	Neg	39.6	na	na	39.9	0.0	Neg	39.7	na	na	40.1	0.0	Neg
Pig 134	39.5	na	na	39.6	na	na	39.6	na	na	39.8	30.7	Neg	41.5	na	na	41.7	19.0	Pos	42.2	na	na	42.0	22.4	Neg	41.6	21.6	Neg			
Pig 139	39.9	0.0	Neg	39.6	na	na	40.2	31.9	Neg	40.4	na	na	41.2	20.6	Pos	41.6	na	na	41.3	21.0	Pos									
Pig 140	39.5	na	na	38.8	na	na	39.3	na	na	39.7	na	na	39.5	23.6	Neg	41.3	na	na	41.4	20.3	Pos									
Pig 141	40.0	0.0	Neg	39.3	na	na	40.7	26.2	Neg	40.6	na	na	41.4	19.9	Pos	41.0	19.8	Pos	na	na	na									
Pig 144	39.6	na	na	39.0	na	na	39.2	0.0	Neg	40.0	na	na	41.5	23.7	Neg	41.1	na	na	41.1	21.6	Pos									
Pig 145	39.6	na	na	39.1	na	na	39.9	35.5	Neg	40.5	na	na	41.0	21.0	Pos	40.5														
Pig 146	39.5	na	na	38.8	na	na	39.8	24.9	Neg	41.4	na	na	40.8	19.7	Pos	41.0	na	na	40.8	21.7	Pos	41.3								

In a recent ASF transmission study, one pig (#138) was directly inoculated (intramuscularly) with ASFV Georgia 2007/1 and introduced into a pen with healthy pigs. The whole blood samples collected every other day from the animals that developed clinical signs were tested by real-time PCR and the PenCheck^®^ LFA. Pos, positive (green); Neg, negative (yellow); Temp., temperature; na, not available (gray). The black cells signify the dpe where the animals died.

**Table 3:** Comparing PenCheck^®^ LFA results with the onset of clinical sign (fever) and detection of ASFV genomic material in ASFV Nigeria RV502-infected pigs.

**A**																						
**DPI 0**			**DPI 1**				**DPI 2**			**DPI 3**			**DPI 4**			**DPI 5**			**DPI 6**			
**Sample**	**Pig #**	**Temp.**	**Ct**	**PenCheck**	**Temp**	**Ct**	**PenCheck**	**Temp**	**Ct**	**PenCheck**	**Temp**	**Ct**	**PenCheck**	**Temp**	**Ct**	**PenCheck**	**Temp**	**Ct**	**PenCheck**	**Temp**	**Ct**	**PenCheck**
**Temperature**	Pig 1	39.5	na	na	39.3	na	na	39.5	na	na	39.6	na	na	40.6	na	na	41.3	na	na	na	na	na
	Pig 2	39.9	na	na	39.5	na	na	39.9	na	na	40.6	na	na	41.3	na	na	41.6	na	na	41.2	na	na
**Whole blood**	Pig 1	na	0.00	Neg	na	0.00	Neg	na	0.00	Neg	na	30.81	Neg	na	21.10	Pos	na	19.37	Pos	na	na	na
	Pig 2	na	0.00	Neg	na	0.00	Neg	na	0.00	Neg	na	0.00	Neg	na	27.12	Pos	na	21.07	Pos	na	20.86	Pos
**Nasal swab**	Pig 1	na	0.00	Neg	na	35.40	Neg	na	0.00	Neg	na	31.80	Neg	na	29.35	Neg	na	22.58	Neg	na	25.76	Neg
	Pig 2	na	0.00	Neg	na	0.00	Neg	na	0.00	Neg	na	0.00	Neg	na	35.39	Neg	na	27.50	Neg	na	24.26	Neg
**Oral swab**	Pig 1	na	0.00	Neg	na	0.00	Neg	na	0.00	Neg	na	0.00	Neg	na	31.82	Neg	na	28.85	Neg	na	25.09	Neg
	Pig 2	na	0.00	Neg	na	0.00	Neg	na	0.00	Neg	na	0.00	Neg	na	34.64	Neg	na	31.07	Neg	na	24.04	Neg
**Rectal swab**	Pig 1	na	0.00	Neg	na	0.00	Neg	na	0.00	Neg	na	0.00	Neg	na	29.21	Neg	na	26.82	Neg	na	26.46	Neg
	Pig 2	na	0.00	Neg	na	0.00	Neg	na	0.00	Neg	na	0.00	Neg	na	na	Neg	na	29.48	Neg	na	24.32	Neg
**Serum**	Pig 1	na	0.00	Neg	na	0.00	Neg	na	0.00	Neg	na	0.00	Neg	na	21.01	Pos	na	na	na	na	na	na
	Pig 2	na	0.00	Neg	na	0.00	Neg	na	0.00	Neg	na	0.00	Neg	na	30.43	Neg	na	na	na	na	18.74	Pos
**B**																						
**Sample**													**Ct Value**					**PenCheck**				
													**ASFV**		**β-Actin**							
Pig 1: SMLN-Right													17.12		18.76			Positive				
Pig 2: SMLN-Right													19.94		18.97			Positive				
Pig 1: SMLN-Left													17.14		18.83			Positive				
Pig 2: SMLN-Left													20.01		18.81			Positive				
Pig 1: SILN-Right													18.07		20.30			Positive				
Pig 2: SILN-Right													21.11		21.24			Positive				
Pig 1: SILN-Left													17.98		20.14			Positive				
Pig 2: SILN-Left													21.93		21.95			Positive				
Pig 1: Tonsil													17.51		19.23			Positive				
Pig 2: Tonsil													22.05		20.96			Positive				
Pig 1: Spleen													18.40		19.57			Positive				
Pig 2: Spleen													17.73		19.00			Positive				

Whole blood, serum, and swab (nasal, oral, and rectal) samples (**A**) and different tissue samples (**B**) collected from two ASFV Nigeria RV502-infected pigs were evaluated for the presence of ASFV genomic material by real-time PCR and to assess the performance of PenCheck^®^ LFA. SMLN: sub-mandibular lymph node; SILN: superficial inguinal lymph node. For (**A**), the β-actin Ct value range for all the samples (positive or negative) tested by real-time PCR was 18.76 to 35.78. Pos, positive (green); Neg, negative (yellow); na, not available (gray).

**Table 4:** Assessing the performance of PenCheck^®^ LFA using field samples.

**A**				
		**Ct Value**		**PenCheck**
**Country**	**Sample Type**	**ASFV**	**B-Actin**	
Ghana (38)	Whole blood (17)	16.13	19.79	Pos
		22.46	25.69	Neg
		29.87	23.78	Pos
		18.14	19.85	Pos
		19.85	21.49	Pos
		39.38	21.86	Neg
		29.76	23.88	Pos
		20.14	21.74	Pos
		20.93	22.39	Pos
		37.86	24.90	Neg
		39.00	26.03	Pos
		36.30	25.16	Neg
		38.85	25.45	Neg
		35.38	26.39	Neg
		20.96	22.41	Pos
		22.50	22.76	Pos
		20.67	24.80	Pos
	Plasma (1)	22.66	21.30	Pos
	Spleen (20)	19.32	18.95	Pos
		25.93	18.82	Pos
		33.91	30.63	Pos
		39.15	18.21	Pos
		26.00	18.38	Pos
		20.04	20.08	Pos
		38.99	31.68	Pos
		18.62	18.36	Pos
		35.25	31.68	Pos
		23.33	23.76	Pos
		34.55	33.24	Pos
		26.06	19.86	Pos
		24.19	23.24	Pos
		31.11	29.04	Neg
		38.45	31.18	Pos
		26.20	19.02	Pos
		32.39	28.85	Pos
		20.77	19.56	Pos
		19.47	19.95	Pos
		26.13	21.05	Pos
Nigeria (12)	Spleen (12)	22.74	21.83	Pos
		22.21	21.70	Pos
		19.94	21.11	Pos
		23.25	22.13	Pos
		19.25	25.44	Pos
		19.85	20.91	Pos
		20.99	21.60	Pos
		22.51	20.26	Pos
		18.74	20.56	Pos
		23.81	26.57	Pos
		23.04	26.09	Pos
		26.72	21.40	Pos
**B**				
**ASFV Real-Time PCR-Negative Samples (No Ct Value)**			**PenCheck**	
			**Negative**	**Positive**
179			178 (99.4%)	1 (0.6%)

Fifty ASFV real-time PCR-positive field samples (whole blood, plasma, and spleen) collected from Ghana and Nigeria were tested on PenCheck^®^ LFA (**A**). In addition, a total of 179 ASFV real-time PCR-negative field whole blood samples (with no Ct values) from Ghana (100) and Canada (79) were tested on PenCheck^®^ LFA (**B**) to assess assay specificity. The β-actin Ct value range for the 179 ASFV real-time PCR-negative field whole blood samples was 20.25 to 30.17.

## Data Availability

The authors will provide the data related to this manuscript upon request.

## Supplementary Materials

The following supporting information can be downloaded at: https://www.mdpi.com/article/10.3390/pathogens11020138/s1. Figure S1. The workflow of PenCheck^®^ LFA testing. To conduct the PenCheck^®^ LFA test, the lyophilized reagents were reconstituted with 200 µL of water, followed by the addition of 10 µL of the sample. The sample mixture was incubated for 5 min at room temperature following gentle swirling. After the 5 min incubation period, the tube was gently swirled, and the test strip was inserted into the vial. The strips were examined by the naked eye for results after 20 min of incubation at room temperature. One line indicates a negative result, while two lines indicate a positive result. Figure S2. Timeline of the experiment described in Table 2. Pig #138 (seeder pig) was infected with 1 × 10^5^ TCID_50_ of ASFV Georgia 2007/1 (in 1 mL) intramuscularly and immediately returned to the pen containing the naïve contact pigs. The ASFV transmission from the seeder pig to the contact pigs was monitored. The animals were monitored for clinical signs, and their rectal temperatures were recorded daily. Whole blood samples were collected for real-time PCR and PenCheck^®^ LFA analysis on the indicated days.
