# Supplementary material for: Evaluation of a Lateral Flow Assay for Rapid Detection of African Swine Fever Virus in Multiple Sample Types
Source: Pathogens. 2022 Jan 24;11(2):138. doi: 10.3390/pathogens11020138 (PMC8877915; doi:10.3390/pathogens11020138)
Supplement: Supplementary file 1 [file pathogens-11-00138-s001.zip › pathogens-1543263-supplementary.pdf]

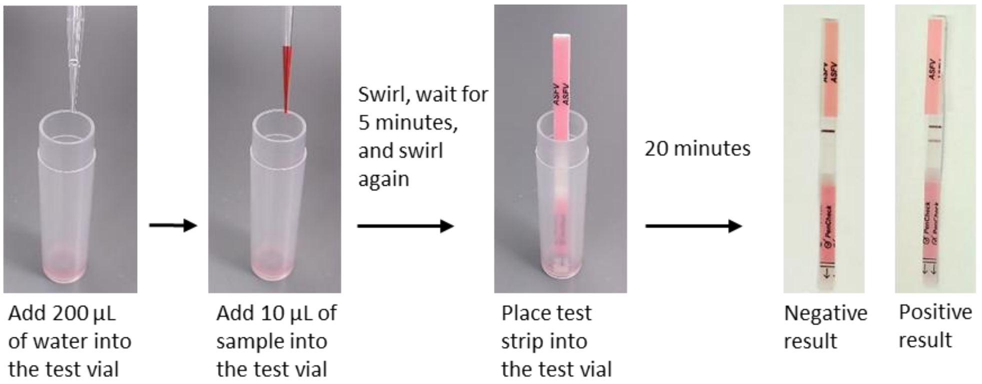

Figure S1. The workflow of PenCheck® LFA testing. To conduct the PenCheck® LFA test, the lyophilized reagents were reconstituted with 200 µL of water, followed by the addition of 10 µL of the sample. The sample mixture was incubated for 5 min at room temperature following gentle swirling. After the 5 min incubation period, the tube was gently swirled, and the test strip was inserted into the vial. The strips were examined by the naked eye for results after 20 min of incubation at room temperature. One line indicates a negative result, while two lines indicate a positive result.

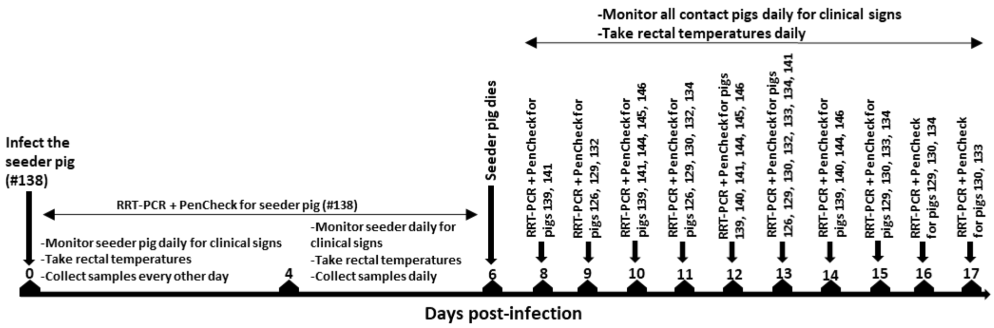

Figure S2. Timeline of the experiment described in Table 2. Pig #138 (seeder pig) was infected with 1X105 TCID50 of ASFV Georgia 2007/1 (in 1 mL) intramuscularly and immediately returned to the pen containing the naïve contact pigs. The ASFV transmission from the seeder pig to the contact pigs was monitored. The animals were monitored for clinical signs, and their rectal temperatures were recorded daily. Whole blood samples were collected for real-time PCR and PenCheck® LFA analysis on the indicated days.
